# Genome-Wide Association Study (GWAS)-Derived Type 2 Diabetes Risk Variants in Gestational Diabetes Mellitus: Evidence From North India

**DOI:** 10.7759/cureus.101780

**Published:** 2026-01-18

**Authors:** Zoya Shakir, Amita Pandey, Abdulrahman A. Alsayegh, Abrar Fahad Alshahrani, Fauzia Ashfaq, Mohammad Khan, Wahid Ali

**Affiliations:** 1 Department of Pathology, King George's Medical University, Lucknow, IND; 2 Department of Obstetrics and Gynaecology, King George's Medical University, Lucknow, IND; 3 Department of Clinical Nutrition, College of Nursing and Health Sciences, Jazan University, Jazan, SAU; 4 Department of Clinical Nutrition, College of Applied Health Sciences, Qassim University, Ar Rass, SAU

**Keywords:** bio-marker, diabetes gestational, genome-wide association study (gwas), snp genotyping, types 2 diabetes

## Abstract

Introduction: Gestational diabetes mellitus (GDM) is hyperglycaemia first identified during pregnancy. Because GDM and type 2 diabetes mellitus (T2DM) share insulin resistance and β-cell dysfunction, T2DM variants may also influence the risk of GDM. This study examined the association of three genome-wide association study (GWAS)-identified T2DM variants, AP3S2 rs2028299 (C>A), ST6GAL1 rs16861329 (C>T), and VPS26A rs1802295 (C>T), with GDM susceptibility in pregnant women from North India.

Methods: This observational case-control study was conducted at the tertiary care antenatal clinic of a medical university in Uttar Pradesh, India, and included 69 women with GDM and 69 age- and BMI-matched non-GDM controls. GDM was diagnosed using a one-step 75 g oral glucose tolerance test (OGTT) according to the Diabetes in Pregnancy Study Group India (DIPSI) criteria (DIPSI ≥140 mg/dL). Genotyping of the three variants was performed using polymerase chain reaction followed by Sanger sequencing.

Results: Women with GDM had higher triglyceride (p=0.037) and VLDL (p=0.006) levels than controls. A family history of T2DM was significantly associated with GDM (p=0.024). The *AP3S2* A allele increased GDM risk 2.26-fold (OR=2.26, 95% CI: 1.27-4.02, p=0.005). The *ST6GAL1* CT genotype (OR=2.30, 95% CI: 1.16-4.58, p=0.017) and T allele (OR=1.95, 95% CI: 1.12-3.41, p=0.018) were also associated with increased GDM risk.

Conclusion: In this North Indian case-control study, rs2028299 (*AP3S2*) and rs16861329 (*ST6GAL1*) were significantly associated with GDM, suggesting potential overlap in genetic susceptibility with T2DM. However, functional studies and larger multi-center cohorts are needed to clarify biological mechanisms and confirm generalizability.

## Introduction

Gestational diabetes mellitus (GDM) has been defined as a condition of hyperglycemia of variable severity, with onset or first recognition during pregnancy [1]. It features impaired glucose tolerance (IGT) and insulin resistance, linked to hormonal imbalances such as human placental lactogen and progesterone during pregnancy, which affect pancreatic beta cell function [2]. In susceptible individuals, inadequate pancreatic β-cell compensation for increased insulin demand leads to hyperglycemia and increased risk of GDM [3].

Globally, diabetes affects nearly one in six pregnancies [4], with 90% linked to GDM. In Southeast Asia, its prevalence is 20.8%, the highest globally, attributed to genetic factors and high-carbohydrate diets [5]. India faces a rapidly increasing GDM prevalence, paralleling rising maternal obesity and advancing maternal age [6]. GDM is clinically significant not only for its immediate obstetric risks but also for its long-term metabolic consequences, including a markedly higher likelihood of progression to T2DM in both mothers and offspring [7]. The strong clinical and metabolic overlap between GDM and T2DM, including β-cell dysfunction, insulin resistance, and shared risk factors such as family history, suggests that the two conditions may also share underlying genetic determinants [8,9].

A genome-wide association study (GWAS) conducted on a cohort of South Asian origin has identified six novel T2DM single-nucleotide polymorphisms (SNPs) at different genetic loci: *GRB14* (rs3923113), *ST6GAL1* (rs16861329), *AP3S2* (rs2028299), *VPS26A* (rs1802295), *HMG20A* (rs7178572), and *HNF4A* (rs4812829) [10]. However, only a limited number of these loci have been assessed for association with GDM [11], and existing studies show considerable regional variation within India and other populations [12]. *HMG20A* and *HNF4A* variants have been associated with GDM in South Indian populations [13], but not in North Indian cohorts [14], whereas *GRB14* shows the opposite trend [13,14]. These discrepancies may be due to variations in allele frequencies between the North and South Indian populations, which are ethnically distinct. These differences highlight the importance of population-specific validation.

 The present study's primary objective was to investigate the association of three South Asian GWAS-identified T2DM variants, *AP3S2* rs2028299, *ST6GAL1* rs16861329, and *VPS26A* rs1802295, with the risk of GDM in a cohort of pregnant women from North India, and the secondary objective was to explore their relationship with metabolic parameters, including serum lipids. This work provides important population-specific genetic insights into the pathogenesis of GDM.

## Materials and methods

Study design

This was a single-centre, observational case-control study conducted at the antenatal clinic (ANC) of the Department of Obstetrics and Gynaecology at King George’s Medical University (KGMU), a tertiary care hospital in Lucknow, Uttar Pradesh, India, from March 2024 to March 2025. The study was approved by the KGMU Institutional Ethics Committee (reference code: 111th ECM II B-Ph.D/P1). Written informed consent was taken from all subjects before enrolment.

Eligibility and sample size

During their first ANC visit, pregnant women were screened for eligibility. The inclusion criteria included ages 18-40 years, a gestational age of 24-28 weeks, and willingness to provide written informed consent. Exclusion criteria encompassed pre-existing diabetes, cardiovascular or cerebrovascular disease, any malignancy, and chronic renal or hepatic disorders.

The sample size was calculated using Cochran’s formula, with a GDM prevalence of 10% [15]. We enrolled a total of 138 pregnant women and divided them into two equal groups: 69 women with GDM and 69 non-GDM (NGDM) controls. Controls were individually matched to GDM cases based on age and body mass index (BMI) to minimise confounding.

GDM diagnosis

GDM was diagnosed in accordance with the Indian National Guidelines (2014), endorsed by the Diabetes in Pregnancy Study Group India (DIPSI). A single-step, non-fasting 75-g oral glucose tolerance test (OGTT) was performed, with a two-hour cutoff≥140 mg/dL (≥7.8 mmol/L) to enable universal, same-visit screening widely adopted in India. Women with an initial non-diagnostic test were retested at 24-28 weeks; persistently normal results were classified as non-GDM (NGDM) [16]. For context, we considered International Association of Diabetes and Pregnancy Study Groups (IADPSG) thresholds (diagnosis if any one of fasting plasma glucose (FPG) ≥92, one-hour plasma glucose ≥180, two-hour plasma glucose ≥153 mg/dL) when identifying clinically meaningful hyperglycemia during interpretation [17,18].

Data collection

A structured questionnaire (see Appendices) was employed to gather demographic information, obstetric history, and family history of diabetes. Additionally, anthropometric measurements such as weight and height were recorded to calculate BMI using the Quetelet formula [19].

Blood Collection and DNA Extraction

Venous blood (5 mL) was collected two hours post-75 g glucose ingestion per DIPSI protocol: 1 mL in a grey-top vacutainer (sodium fluoride and potassium oxalate) for two-hour plasma glucose measurement, 2 mL in an EDTA-coated vacutainer (BD Vacutainer®, Becton, Dickinson and Company, Franklin Lakes, New Jersey, United States) for genomic DNA extraction, and 2 mL in a plain red-top vacutainer for serum separation. After clotting, the samples were centrifuged at 1500 rpm for 15 minutes to facilitate lipid profile analysis. Biochemical parameters, including total cholesterol (TC), triglycerides (TG), and high-density lipoprotein (HDL), were analysed using the Transasia XL-300 automated analyser. Low-density lipoprotein (LDL) was calculated using the modified Friedewald formula, and very low-density lipoprotein (VLDL) was determined enzymatically.

DNA was extracted from EDTA-anticoagulated blood leukocytes using OmniPrep™ Genomic DNA Kit (Catalogue No. 786-136; Geno Technology, Inc. (G-Biosciences), St. Louis, Missouri, United States). DNA was stored in DNase-free tubes at -20 °C, quantified by 1% agarose gel electrophoresis, and purity was determined by spectrophotometric A260/A280 ratio using a NanoDrop (Thermo Fisher Scientific Inc., Waltham, Massachusetts, United States).

In-Silico Primer Design and Genotyping

Flanking regions of rs2028299, rs1802295, and rs16861329 were retrieved from Ensembl Genome Browser (V113) (http://www.ensembl.org/). Basic Local Alignment Search Tool (BLAST) verified sequence specificity. Primers were created with the PrimerQuest™ tool from Integrated DNA Technologies, Inc. (Coralville, Iowa, United States). Primer characteristics, including GC content, melting temperature, and secondary structure propensity, were evaluated using the Sequence Manipulation Suite (V2). In the final step, the designed primers were assessed using the In-Silico PCR tool from the UCSC Genome Browser (http://genome.ucsc.edu/) to confirm target-specific amplification and exclude off-target binding. This multistep strategy facilitated the generation of primers with high specificity and suitability for Sanger sequencing.

Genotyping of rs2028299 (C>A) located near the *AP3S2*, rs1802295 (C>T) near the *VPS26A*, and rs16861329 (C>T) near the *ST6GAL1* was amplified by polymerase chain reaction (PCR), followed by Sanger sequencing of the resulting amplicons. Loci were amplified using the SureCycler™ 8800 Thermal Cycler with a 96-well, inter-exchangeable block (Agilent Technologies, Inc., Santa Clara, California, United States). The PCR was run at 94°C for five minutes, followed by 35 cycles of denaturation at 94°C for 45 seconds, annealing at 58°C for 30 seconds, and extension at 72°C for 45 seconds. A final extension was performed at 72°C for seven minutes.

**Figure 1:**
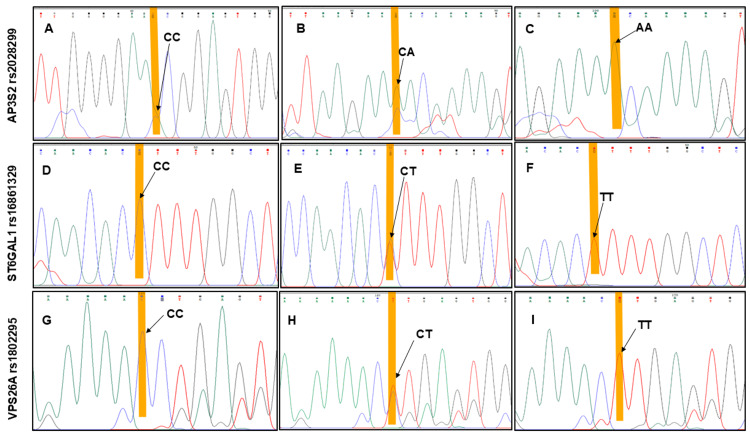
Representative sequence chromatograms of AP3S2, ST6GAL1, and VPS26A genotypes. Panel A–C: Chromatograms showing the CC, CA, and AA genotypes of AP3S2 rs2028299. Panel D–F: Chromatograms showing the CC, CT, and TT genotypes of ST6GAL1 rs16861329. Panel G-I: Chromatogram showing the CC, CT, and TT genotype of VPS26A rs1802295. Highlighted variants indicate the nucleotide substitutions.

Reactions (30 µL) contained 1-2 µL genomic DNA, 1.5 µL each primer, 15.5 µL Taq DNA Polymerase Master Mix (Thermo Fisher Scientific Inc.), and 10.8 µL nuclease-free water. Amplicons were resolved on a 1.5% agarose gel stained with ethidium bromide, alongside a 100 bp DNA ladder (Thermo Fisher Scientific Inc.), and visualised on a Gel Doc™ EZ Imager (Bio-Rad Laboratories, Inc., Hercules, California, United States). Primer pairs yielded fragments of 295 bp (*AP3S2*: F 5′-CTCAGGAAGACATCCCTAACAC-3′; R 5′-TCAGTGGAAGTTGGCAGATAC-3′), 396 bp (*ST6GAL1*: F 5′-TGTGTGTCTGTGTGTGTATG-3′; R 5′-GGTGGCTGGATGTGTTCTTA-3′), and 522 bp (*VPS26A*: F 5′-GGAACTTTGTTAAGCTGCCTTT-3′; R 5′-CACACAACGGTACTACTGGAATA-3′). PCR products were purified and submitted to the Sanger sequencing facility for genotyping. Sequencing data were analysed using Chromas software version 2.6.6 (Technelysium Pty Ltd, Brisbane, Queensland, Australia) to interpret chromatograms and confirm the presence of the respective alleles (Figure 1).

Statistical analysis

All statistical analyses were performed using the IBM SPSS Statistics for Windows, version 19 (Released 2010, IBM Corp., Armonk, New York, United States). The student’s t-test was utilised to compare quantitative variables between the GDM and NGDM groups. One-way ANOVA was used to analyse the association between genotypes and lipid parameters, as well as other continuous traits. The associations of *VPS26A, AP3S2*, and *ST6GAL1* genotypes with GDM were evaluated by calculating odds ratios (ORs) with 95% confidence intervals (CIs). Allele and genotype frequencies were determined using gene counting and compared between cases and controls using the Chi-square (χ²) test. Hardy-Weinberg equilibrium (HWE) for genotype distributions was assessed using the χ² test. A p-value of less than 0.05 was considered statistically significant.

## Results

Demographic and clinical characteristics

Among the 138 women included, age and BMI were comparable between the GDM and NGDM groups (Table 1). GDM cases had significantly higher triglyceride levels (p = 0.037), VLDL levels (p = 0.006), gestational age (p = 0.010), and a higher frequency of family history of T2DM (p = 0.024), supporting a familial metabolic susceptibility to GDM [20]. DIPSI values were significantly elevated in the GDM group (p < 0.001). The prevalence of ≥2 miscarriages/stillbirths was higher among GDM women (p = 0.042).

**Table 1:** Demographic and clinical characteristics of non-GDM (NGDM) compared to gestational diabetes mellitus (GDM) groups (n = 138). Values are presented as mean ± SD for continuous variables and n (%) for categorical variables. Comparisons between groups were made using the Pearson chi-square (χ²) test (categorical) or unpaired t-test (continuous). A p-value < 0.05 was considered statistically significant. BMI: body mass index; GDM: gestational diabetes mellitus; NGDM: non-GDM; T2DM: type 2 diabetes; SBP: systolic blood pressure; DBP: diastolic blood pressure; DIPSI: Diabetes in Pregnancy Study Group India; HDL: high-density lipoprotein; LDL: low-density lipoprotein; VLDL: very-low-density lipoprotein

Parameters	NGDM group (n=69)	GDM group (n=69)	Chi-square / t-value	p-value
Demographics				
Age (years), mean±SD	27.46 ± 5.07	28.23 ± 4	4.85	0.183
Gestational Age (weeks), n (%)				
≤24–28	23 (33.3)	38 (55.1)	6.61	0.010
>28	46 (66.7)	31 (44.9)		
BMI (kg/m²), n (%)				
<18.5	3 (4.3)	5 (7.2)	1.61	0.657
18.5–24.9	31 (44.9)	26 (37.7)		
25.0–29.9	23 (33.3)	28 (40.6)		
≥30.0	12 (17.4)	10 (14.5)		
Family history, n (%)				
Positive T2DM history	14 (20.3)	26 (37.7)	5.07	0.024
Negative T2DM history	55 (79.7)	43 (62.3)		
Obstetrics history				
No. of Stillbirth/Miscarriage, n (%)				
Nil	43 (62.3)	36 (52.2)	6.33	0.042
1	20 (29.0)	16 (23.2)		
>1	6 (8.7)	17 (24.6)		
Clinical parameters, mean±SD				
Systolic Blood Pressure	112.03 ± 12.18	113.75 ± 13.40	0.80	0.425
Diastolic Blood Pressure	71.57 ± 7.13	71.42 ± 9.43	0.06	0.951
Biochemical parameters, mean±SD				
DIPSI test (mg/dl)	112.43 ± 18.93	158.93 ± 22.58	13.11	<0.001
Total Cholesterol (mg/dl)	180.47 ± 57.12	194.81 ± 45.98	1.62	0.107
Triglycerides (mg/dl)	157.05 ± 97.35	189.68 ± 84.21	2.11	0.037
HDL (mg/dl)	63.28 ± 24.47	68.95 ± 18.21	1.55	0.125
LDL (mg/dl)	90.61 ± 24.82	95.70 ± 26.77	1.16	0.248
VLDL (mg/dl)	29.93 ± 17.09	37.80 ± 16.17	2.78	0.006
Serum LDL/HDL (mg/dl)	1.56 ± 0.41	1.46 ± 0.44	-1.28	0.204

Association of *AP3S2* rs2028299 polymorphism with GDM risk

**Figure 2:**
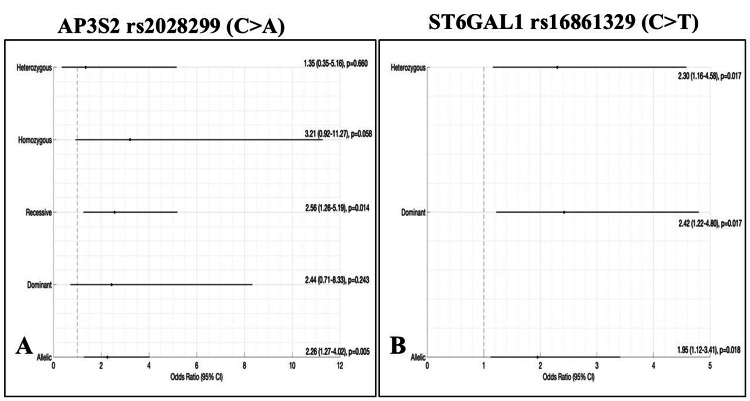
Genetic model associations of AP3S2 rs2028299 and ST6GAL1 rs16861329 with gestational diabetes risk. (A) Forest plot of *AP3S2* rs2028299 (C>A) and (B) Forest plot of *ST6GAL1* rs16861329 (C>T) showing odds ratios (OR) with 95% confidence intervals (CI) under heterozygous, homozygous, recessive, dominant, and allelic models.

Genotypic and allelic distributions of AP3S2 rs2028299 in NGDM controls and GDM cases are presented in Table 2. The A allele was significantly more common in GDM cases, conferring a 2.26-fold increased risk (p = 0.005). Under the recessive model (AA vs. CA+CC), the AA genotype was associated with a 2.56-fold increased risk of GDM (p = 0.014). No significant association was observed under dominant or heterozygous models (Figure 2A).

**Table 2:** Risk analysis of AP3S2, ST6GAL1, and VPS26A gene variants in women with gestational diabetes (GDM) and NGDM controls (n=138). Associations were analysed by the Pearson chi-square test (χ2). A p-value < 0.05 was considered statistically significant. SNP: single-nucleotide polymorphism; GDM: gestational diabetes mellitus; NGDM: non-gestational diabetes mellitus

Gene/SNPs Chr: Position	Genotype	NGDM (n= 69), n (%)	GDM (n= 69), n (%)	OR (95% CI)	(χ2)	p-value
*AP3S2* rs2028299 C>A Chr (GRCh38): 15:89,831,025.	CC	9 (13)	4 (5.8)	Ref.	-	-
	CA	25 (36.2)	15 (21.7)	1.35 (0.35–5.16)	0.19	0.660
	AA	35 (50.7)	50 (72.5)	3.21 (0.92–11.27)	3.59	0.058
	C (Allele)	43 (31.2)	23 (16.7)	Ref.	-	-
	A (Allele)	95 (68.8)	115 (83.3)	2.26 (1.27–4.02)	7.97	0.005
*ST6GAL1* rs16861329 C>T Chr (GRCh38): 3:186,948,673	CC	43 (62.3)	28 (40.6)	Ref.	-	-
	CT	26 (37.7)	39 (56.5)	2.30 (1.16–4.58)	5.74	0.017
	TT	0 (0)	2 (2.9)	-	2.95	0.086
	C (Allele)	112 (81.2)	95 (68.8)	Ref.	-	-
	T (Allele)	26 (18.8)	43 (31.2)	1.95 (1.12–3.41)	5.59	0.018
*VPS26A* rs1802295 C>T Chr (GRCh38): 10:69,171,718.	CC	11 (15.9)	13 (18.8)	Ref.	-	-
	CT	48 (69.6)	41 (59.4)	0.72 (0.29-1.79)	0.497	0.481
	TT	10 (14.5)	15 (21.7)	1.27 (0.41-3.94)	0.170	0.680
	C (Allele)	70 (50.7)	67 (48.6)	Ref.	-	-
	T (Allele)	68 (49.3)	71 (51.4)	1.09 (0.68-1.75)	0.058	0.810

Association of *ST6GAL1* rs16861329 polymorphism with GDM risk

Genotypic and allelic distributions of *ST6GAL1* rs16861329 in NGDM controls and GDM cases are summarised in Table 2. The CT genotype was significantly associated with GDM (OR = 2.30; p = 0.017). The T allele conferred approximately two-fold higher risk (OR = 1.95; p = 0.018). Dominant-model (CT+TT vs. CC; p = 0.017) analysis remained significant. No association was observed under the recessive model due to low TT frequency (Figure 2B).

Association of *VPS26A* rs1802295 polymorphism with GDM risk

No significant association was detected across all genotype or allelic comparisons, suggesting no role of this locus in GDM susceptibility in this population.

Genotype association with DIPSI and lipid parameters

*AP3S2* rs2028299 showed a significant association with DIPSI values (p = 0.002), with the AA genotype having the highest mean glucose levels. No SNP demonstrated significant associations with lipid parameters, indicating that these variants may influence glucose metabolism independent of lipid pathways (Table 3).

**Table 3:** Association of AP3S2, ST6GAL1 and VPS26A genotypes with DIPSI and lipid parameters One-way ANOVA was performed to assess the association between *AP3S2* (CC, CA, AA) and *ST6GAL1* (CC, CT, TT) genotypes with DIPSI and lipid profile parameters. The model included genotype as the fixed factor and each biochemical parameter as the dependent variable. Effect size is reported as partial eta squared (η²). A p-value < 0.05 was considered statistically significant. DIPSI: Diabetes in Pregnancy Study Group India; TG: triglycerides; HDL: high-density lipoprotein; LDL: low-density lipoprotein; VLDL: very-low-density lipoprotein

Genotype	DIPSI (mg/dL)	Cholesterol (mg/dL)	TG (mg/dL)	HDL (mg/dL)	LDL (mg/dL)	VLDL (mg/dL)	LDL/HDL (mg/dL)
AP3S2							
CC, mean±SD	129.23 ± 25.84	193.72 ± 67.79	166.78 ± 83.19	65.29 ± 22.13	95.14 ± 33.20	32.25 ± 14.15	1.61 ± 0.57
CA, mean±SD	122.57 ± 28.85	187.48 ± 52.24	169.96 ± 101.41	65.01 ± 19.91	94.92 ± 28.61	32.70 ± 17.59	1.54 ± 0.40
AA, mean±SD	142.83 ± 31.13	186.79 ± 50.07	175.98 ± 89.82	66.75 ± 22.61	92.02 ± 23.43	34.66 ± 17.31	1.48 ± 0.42
F-value	6.51	0.10	0.09	0.10	0.21	0.24	0.60
p-value	0.002	0.906	0.911	0.908	0.809	0.786	0.553
Effect size	0.088	0.001	0.001	0.001	0.003	0.004	0.009
ST6GAL1							
CC, mean±SD	132.45 ± 33.12	193.68 ± 54.71	186.98 ± 95.89	70.17 ± 24.38	93.71 ± 24.53	36.08 ± 17.36	1.46 ± 0.43
CT, mean±SD	138.53 ± 29.22	180.64 ± 49.03	156.89 ± 86.14	61.64 ± 17.60	92.33 ± 27.56	31.10 ± 16.41	1.56 ± 0.42
TT, mean±SD	157.50 ± 0.71	201.20 ± 57.13	225.50 ± 92.63	67.25 ± 21.14	100.26 ± 24.85	45.10 ± 18.53	1.51 ± 0.11
F-value	1.14	1.13	2.17	2.69	0.12	1.92	1.04
p-value	0.323	0.326	0.118	0.071	0.884	0.151	0.356
Effect size	0.017	0.016	0.031	0.038	0.002	0.028	0.015
VPS26A							
CC, mean±SD	155.54 ± 13.71	189.02 ± 43.76	190.25 ± 88.63	66.86 ± 19.55	92.95 ± 42.21	38.05 ± 17.73	1.45 ± 0.38
CT, mean±SD	161.8 ± 26.83	198 ± 45.69	192.69 ± 88.03	68.86 ± 17.43	98.09 ± 27.18	38.09 ± 16.50	1.51 ± 0.45
TT, mean±SD	154.07 ± 13.97	191.12 ± 50.88	180.98 ± 73.92	71.02 ± 20.13	91.53 ± 28.74	36.80 ± 14.87	1.36 ± 0.45
F-value	0.82	0.24	0.10	0.18	0.41	0.04	0.68
p-value	0.444	0.244	0.901	0.836	0.667	0.964	0.506
Effect size	0.024	0.007	0.003	0.005	0.012	0.001	0.02

## Discussion

This study provides novel evidence that the genetic associations of *AP3S2* (rs2028299) and *ST6GAL1* (rs16861329), two SNPs previously identified through GWAS as risk determinants of T2DM in South Asian populations, are also associated with GDM in a North Indian cohort. To our knowledge, this is the first report to demonstrate an association in North Indian women, highlighting a potential shared genetic basis between GDM and T2DM.

*AP3S2* encodes a component of the AP-3 complex that mediates vesicle formation and intracellular trafficking [21]. In β-cells, *AP3S2* regulates insulin granule movement to the plasma membrane and coordinates vesicle docking for glucose homeostasis [22]. Dysfunctional *AP3S2* activity, potentially driven by the rs2028299 polymorphism, could impair granule exocytosis, leading to diminished first-phase insulin secretion and β-cell dysfunction. These defects are significant during pregnancy, as pancreatic islets respond by increasing β-cell proliferation to handle the increased metabolic demands [23]. Impaired vesicle trafficking and defective exocytosis occur in dysglycaemia and are key to T2DM and GDM pathology [24].

The association between *AP3S2* rs2028299 and the risk of GDM has been investigated in numerous research papers [10]. However, no study has reported an association of rs2028299 with GDM risk in their respective populations. In the present study, the intergenic variant rs2028299 (C>A), located near the *AP3S2* gene, may act as a significant genetic marker associated with GDM in the North Indian pregnant women, pending replication in a larger cohort. Under the recessive model, women carrying the AA genotype had a 2.6-fold higher risk of GDM than those with the CA/CC genotype (OR = 2.56, 95% CI: 1.26-5.19; p = 0.014). Similarly, in the allelic model, the A allele conferred a 2.3-fold increased risk of GDM compared with the C allele (OR = 2.26, 95% CI: 1.27-4.02; p = 0.005). These findings align with GWAS evidence linking AP3S2 to T2DM risk in South Asian (OR=1.11; 95% CI: 1.05-1.16) [10], North Indian (OR=2.78; 95% CI:1.55-4.98) [25], and East Asian (OR = 9.50; 95% CI: 5.67-15.94) populations [23]. Thus, it may be hypothesised that the A allele of rs2028299 confers susceptibility to GDM.

*ST6GAL1* encodes a key enzyme responsible for the sialylation of glycoproteins and glycolipids, a post-translational modification critical to cell signalling, immune regulation, and metabolic homeostasis [26]. Mechanistically, ST6GAL1-mediated sialylation influences insulin receptor sensitivity, inflammatory signalling, and glycoprotein stability [27], thereby aggravating insulin resistance during pregnancy. Elevated circulating sialic acid has been reported to correlate with T2DM complications, such as poor glycemic control [28]. Since inflammation and insulin resistance are shared features of T2DM and GDM, these mechanisms provide a plausible link between altered sialylation and increased susceptibility to GDM.

The rs16861329 (C>T) polymorphism, located near the *ST6GAL1* gene, has previously been implicated with T2DM in multiple populations (South Asian: p=0.02; North Indian: p= 0.018) [10,25]. To date, no study has reported a direct or population-specific association between rs16861329 and the risk of GDM. The present investigation found significant associations under the heterozygous (CT vs. CC; OR = 2.30, 95% CI: 1.16-4.58; p = 0.017), dominant (CT+TT vs. CC; OR = 2.42, 95% CI: 1.22-4.80; p = 0.017), and allelic (T vs. C; OR = 1.95, 95% CI: 1.12-3.41; p = 0.018) models. These findings provide novel evidence that *ST6GAL1* rs16861329 could be associated with GDM susceptibility in a North Indian population.

The *VPS26A* gene is vital for retrograde protein transport from endosomes to the trans-Golgi network [29]. In line with our previous findings in T2DM patients [25], no significant association was detected in the current GDM cohort. Additionally, the relationship between variants in *AP3S2*, *ST6GAL1*, and *VPS26A* and lipid parameters was evaluated in patients with GDM.

Limitations

This study is limited by a modest sample size, which may have reduced statistical power for rare homozygous genotypes (e.g., *ST6GAL1* TT), the restriction to a single North Indian cohort, limiting generalizability, the lack of functional validation studies, and the absence of replication in an independent cohort. Nevertheless, the study was adequately powered to detect significant associations, supporting the robustness of the findings. Future research in larger, multi-ethnic populations, complemented by functional assays, will be essential to confirm and extend these findings.

## Conclusions

To our knowledge, this is among the first evaluations of *AP3S2* rs2028299 and *ST6GAL1* rs16861329 polymorphisms, which are significantly associated with an increased risk of GDM in North Indian pregnant women. These variants may have potential utility as genetic risk markers, but larger studies are needed before clinical application. Clinically, this study could offer valuable insights for the early identification of women at elevated risk of GDM. Incorporating genetic screening into early risk assessment, particularly within public health programs in low-resource settings, could guide tailored interventions, improve maternal-fetal outcomes, and reduce long-term healthcare costs.

## Figures and Tables

**Figure 3:**
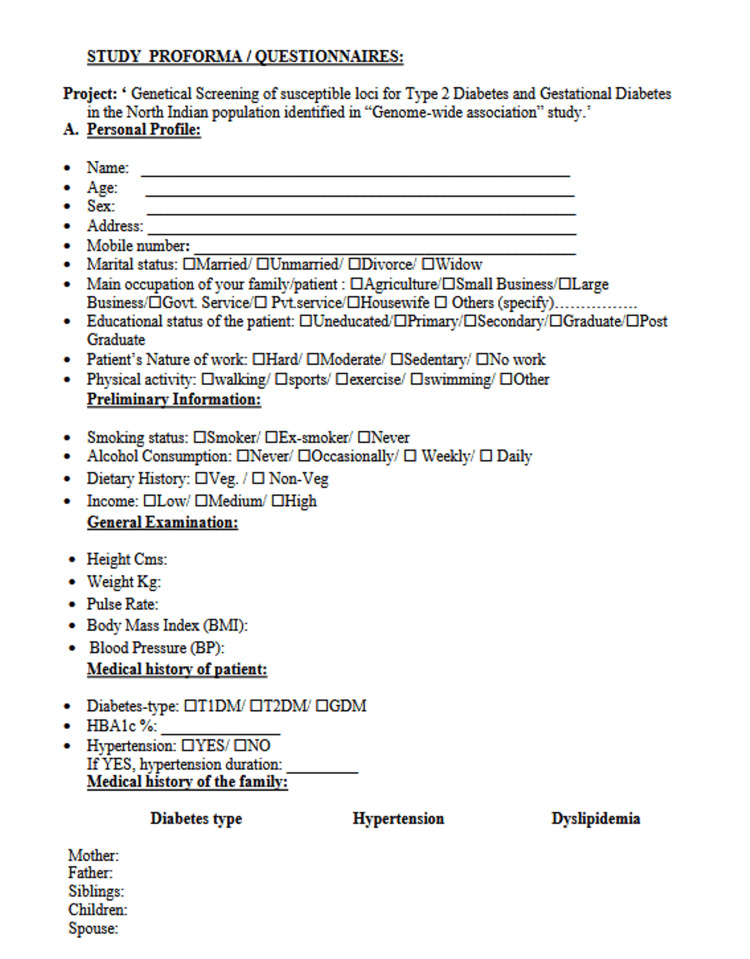
Questionnaire page 1

**Figure 4:**
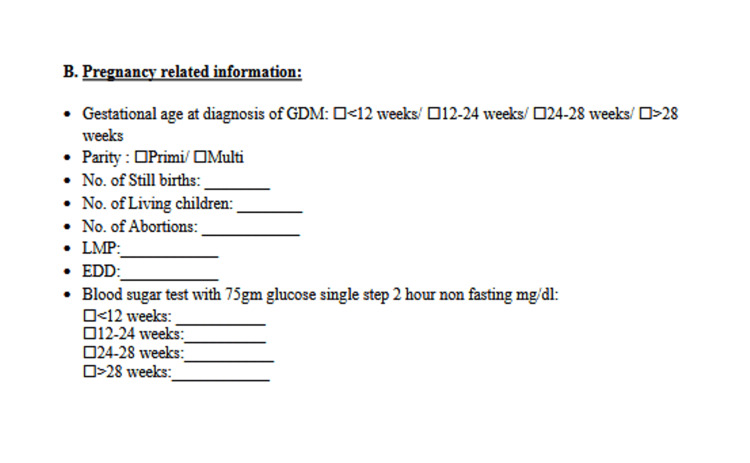
Questionnaire page 2

## Appendices

Study questionnaire 
